# Fetal outcomes after intentional ingestion of paraquat: A case report

**DOI:** 10.1097/MD.0000000000018136

**Published:** 2020-01-03

**Authors:** Jianshi Chen, Xiangdong Jian, Guangcai Yu, Min Si, Baotian Kan

**Affiliations:** a Department of Poisoning and Occupational Diseases, Qilu Hospital of Shandong University, Jinan, Shandong; b Department of Intensive Care Unite, The Second Affiliated Hospital and Yuying Children's Hospital of Wenzhou Medical University, Wenzhou, Zhejiang, PR China.

**Keywords:** fetus, paraquat poisoning, placenta, pregnant woman

## Abstract

**Rationale:**

Despite the fact that treatment of paraquat poisoning in pregnant women and their fetuses is challenging and raises ethical issues, it is rarely reported in the literature. We report the case of a pregnant woman who took paraquat intentionally.

**Patient concerns:**

A 36-year-old woman at 38^+^ weeks gestational age, in an apparent suicide attempt, drank 1 mouthful (about 20 ml) of paraquat solution. Ten hours later, her urine dithionate test showed light blue color with a plasma paraquat concentration of 0.547 μg/ml. Six hours after admission, a male infant, whose plasma paraquat concentration was 0.761 μg/ml, together with 0.673 μg/ml in the amniotic fluid measured by high-performance liquid chromatography, was delivered but the woman's lung, liver, and kidney function declined rapidly.

**Diagnosis:**

1. Acute paraquat poisoning;
2. Third trimester of pregnancy (38^+^ weeks);
3. Placenta previa.

**Interventions:**

Because of placenta previa and multiple organ failure, emergency cesarean section, and panhysterectomy were performed for the pregnant woman. Intravenous injection of antibiotic to prevent infection and dexamethasone 30 mg once a day were administered. Mechanical ventilation was performed for the infant and meropenem and penicillin injection was administered.

**Outcomes:**

The infant died 33 hours after birth while the mother died on the 3rd day after ingestion.

**Lessons:**

Paraquat can enter the fetus through the placenta and the amniotic fluid via fluid exchange. The pathological changes of fetal organs may relate to gestational age, and the prognosis was very poor in both the mother and the fetus.

## 1 Introduction

Paraquat (PQ), also known as Gramoxone or Viologen, is a non-selective herbicide which has been widely used in agricultural production in developing countries. Although most countries have banned it, the number of cases of paraquat poisoning has not decreased significantly. Most cases of paraquat poisoning are due to self-poisoning by oral suicide and the mortality is as high as 20% to 78%.^[1–3]^ Paraquat poisoning in pregnant women and their fetuses is challenging to treat and raises ethical issues, and are rarely reported in the literature. We report a case of intentional paraquat ingestion in a pregnant woman.

## 2 Case report

A 36-year-old pregnant woman with a history of paranoid schizophrenia for more than 10 years and syphilis for 2 years presented at 38^+^ weeks gestational age having drank 1 mouthful (about 20 ml) of paraquat solution 10 hours earlier in an apparent suicide attempt. Before admission to our hospital, the patient was taken to the local hospital for gastric lavage. On admission, the patient had a burning sensation in the throat accompanied by nausea and vomiting with blood but had no chest distress, dyspnea or palpitations, abdominal pain, or vaginal bleeding. However, 4 hours after admission, chest tightness, shortness of breath, and dyspnea were observed.

On initial evaluation, her body weight was 55 kg and vital signs were as follows: temperature 37.1°C, heart rate (HR) 115 beats/minute, respiration (RR) 25 per minute, blood pressure 132/70 mm Hg, and oxygen saturation 98% on room air. Physical examination showed that she was clear minded but had several visible ulcers on the tongue and oral mucosa. Moreover, her heart and lungs were normal and there was no jaundice of the skin or sclera. An abdominal examination showed absence of tenderness and a third trimester pregnancy, with the uterine height being consistent with the estimated gestational age. Babinski sign showed negative results.

**Figure 1:**
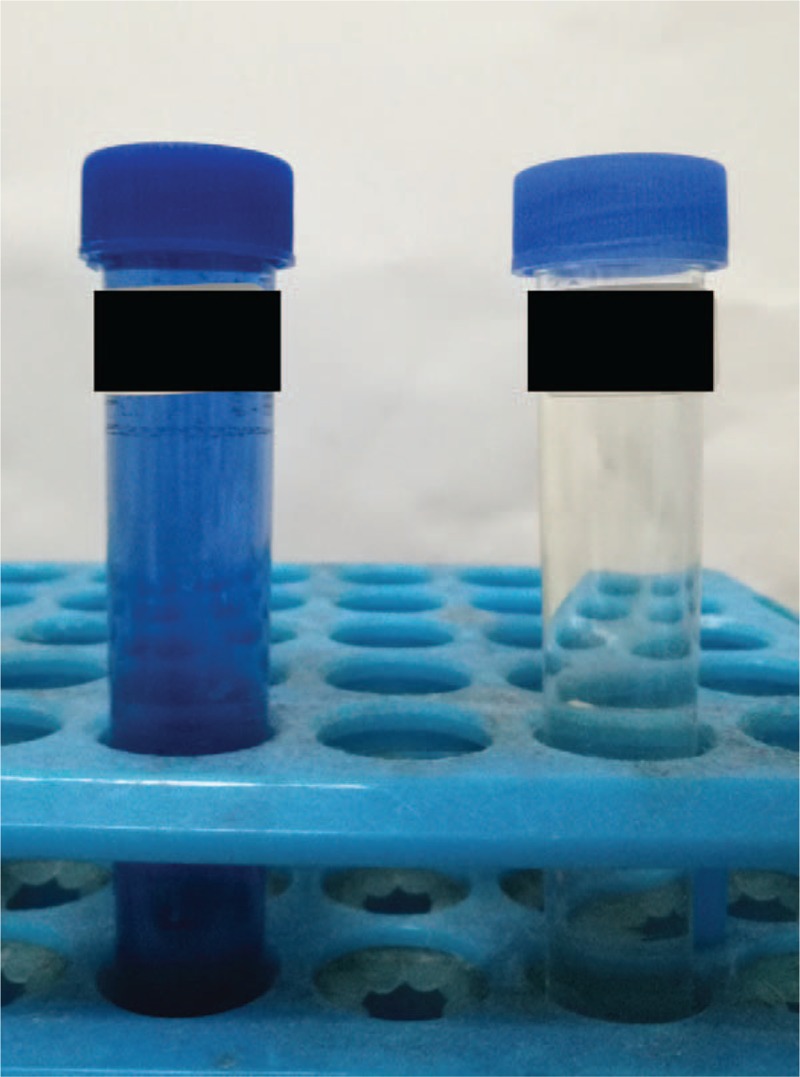
Urine dithionate test showed light blue color (right is contrast).

Paraquat level in blood determined by high performance liquid chromatography (HPLC) at the 10th hour of poisoning was 0.547 μg/ml and the urine dithionate test showed light blue color (Fig. 1). Initial laboratory tests revealed the following: white blood count 19,290 cells/μl (84% neutrophils), hemoglobin 9.3 g/dl, and platelets 231,000/ml. Serum chemistry showed high creatinine 138 μmol/L (normal: 46–106 μmmol/L), uric acid (UA) 456 μmmol/L (normal: 149–446 μmmol/L), aspartate transaminase (AST) 46 μ/L (normal 14–36 u/L), creatine kinase (CK) 399 μ/L (normal: 30–135 μ/L), creatinine kinase MB isoenzyme (CK-MB) 14.5 ng/ml (normal: 0.3–4 ng/ml), lactate dehydrogenase (LDH) 634 u/L (normal: 313–618 μ/L), amylase 221 mmol/L (normal: 30–110 mmol/L). However, total bilirubin (TBIL) level was within the normal range. Arterial blood gas analysis on room air revealed the following: partial pressure of oxygen (PO_2_) 125 mm Hg, partial pressure of carbon dioxide (PCO_2_) 20 mm Hg, Lactic acid 1.6 mmol/L, buffer excess (BE) −11.2 mmol/L, bicarbonate (HCO3^−^) 12.1 mmol/L. However, after 6 hours, creatinine, AST, and TBIL had risen to 192 μmmol/L, 68 μ/L, and 36 μmol/L, respectively, which suggested a deterioration in liver and kidney function.

**Figure 2:**
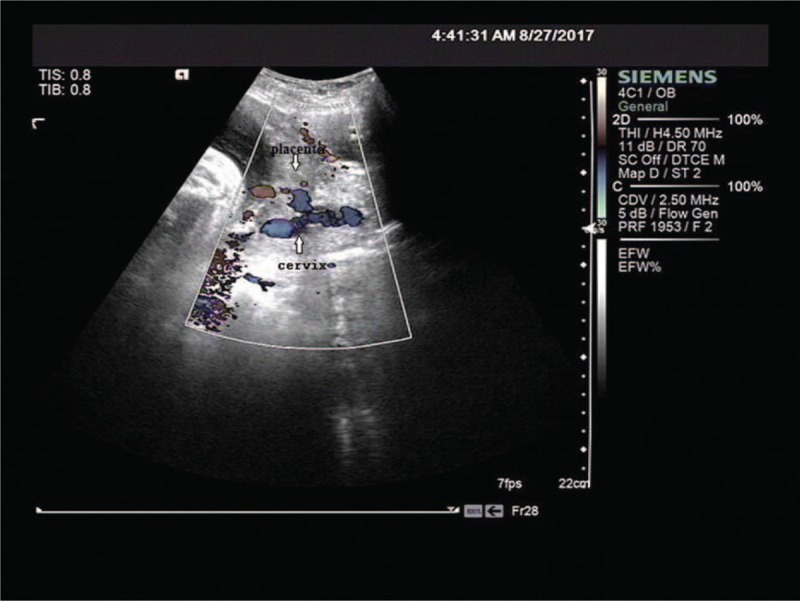
Placenta located in the front wall of uterus and its lower margin covers the cervix.

Without prior antenatal examination, the patient received a prenatal ultrasound which showed an intrauterine singleton pregnancy in breech presentation with a bi-parietal diameter (BPD) of 91 mm and femur length (FL) of 61 mm. Worse still, the placenta was located in the front wall of the uterus with its lower margin covering the cervix, grade I^+^ (Fig. 2). In addition, low echoes in the parenchyma of placenta had also been explored. The fetal heart sounds were audible with a rate of approximately 142 beats/minute and the amniotic fluid was 6.4 cm but had poor internal sound.

## 3 Treatment of the pregnant woman

Due to the pernicious placenta previa, hypoxia partial pressure of 60 mm Hg and poor coagulation function, emergency caesarean section, and panhysterectomy under general anesthesia were performed. During the surgery, the anterior wall of the uterus was covered with varicose veins and about 600 ml yellowish green amniotic fluid with III-degree pollution was found. The location of the placenta was found as described by the ultrasound.

After the surgery, the patient was supported by a ventilator, intravenous dexamethasone 30 mg once a day, and injection of ceftazidime combined with metronidazole to prevent her from contracting infections. Blood transfusion was also performed to rectify bleeding and coagulation function. On the 3rd day after ingestion, she was discharged voluntarily and died within 5 hours of being discharged from the hospital.

## 4 Treatment of the infant

**Figure 3:**
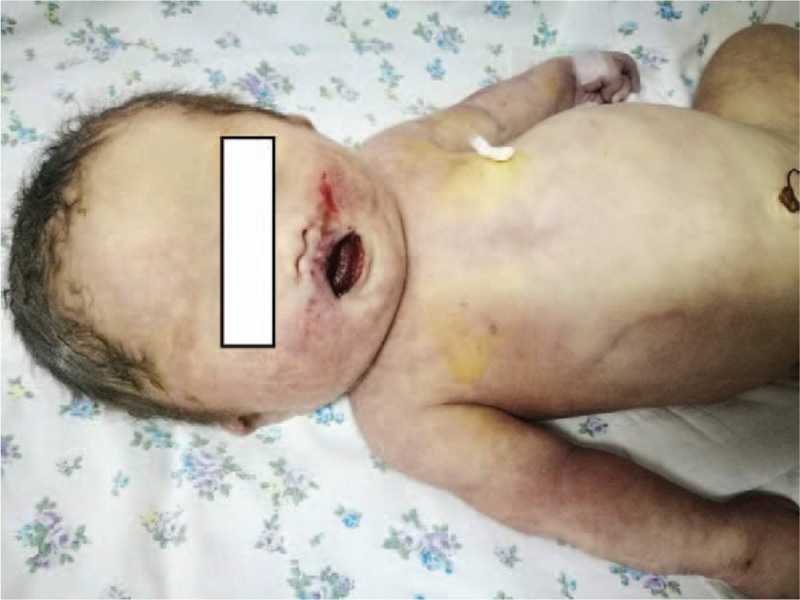
Several ulcers and bleeding points on tongue and oral mucosa.

A male infant weighing 2,400 g, was born via caesarean section approximately 16 hours after the ingestion. Physical examination displayed (Fig. 3) several ulcers and bleeding points on tongue and oral mucosa, wide eye distance, small eye fissure, flat nose bridge, simian line, 3 depressions sign with scattered moist rales in lungs. Endotracheal intubation was performed immediately as his Apgar score was 2 in the first minute, 5 in the fifth minute and 7 in the tenth minute 5 and 710. Subsequently, the infant was transferred to the neonatal intensive care unit (NICU) to receive mechanical ventilation and injection of meropenem and penicillin as a result of being diagnosed with severe neonatal asphyxia, pneumonia, low birth weight (possibly with neonatal hypoxic ischemic encephalopathy), congenital syphilis, or Down syndrome.

**Figure 4:**
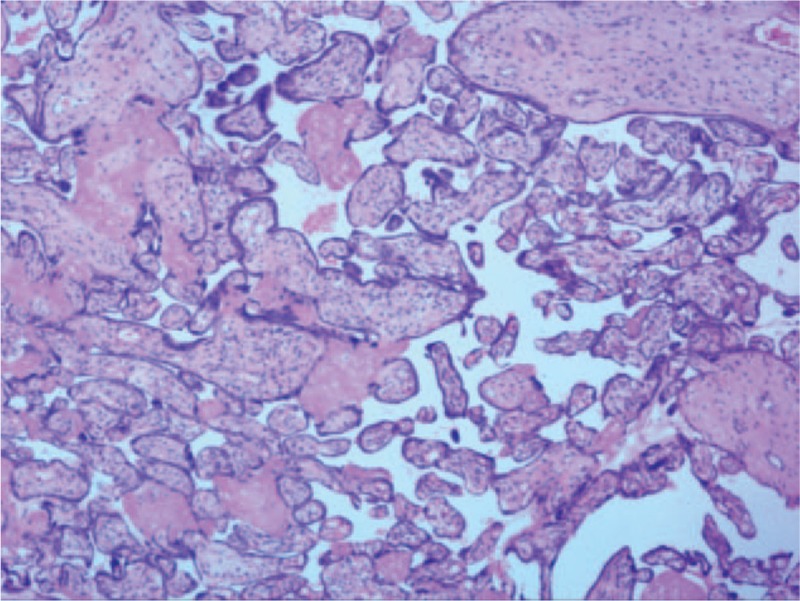
Focal fibrinous necrosis in the placenta.

The laboratory tests of the infant revealed the following: white blood count 8730 cells/μl (47.2% neutrophils), hemoglobin 195 g/dl, and platelets 213,000/ml. Serum chemistry included creatinine 252 μmol/L (normal: 21–75 μmol/L), alanine aminotransferase (ALT) 12 μ/L (14–36 μ/L), AST 63 μ/L (15–40 μ/L), gamma-glutamyl transferase (GGT) 391 μ/L (10–60 μ/L), CK-MB 6.9 ng/ml (0.3–4 ng/ml), TBIL 40.4 μmol/L (5.0–21.0 μmol/L), Indirect bilirubin (IBIL) 37.3 μmol/L (2–15 μmol/L) all of which were suggestive of hepatic and kidney dysfunction. Except phosphorus level of 4.58 mmol/L (0.6–1.6 mmol/L), all other electrolytes were within normal range and the treponema pallidum hemagglutination assay (TPHA) was positive (+). Unfortunately, he died 33 hours after birth. Afterwards, the concentration of paraquat in blood was 0.761 μg/ml and in the amniotic fluid was 0.673 μg/ml. Pathological report showed focal fibrinous necrosis in the placenta (Fig. 4).

## 5 Discussion

Since the first synthesis of paraquat in 1882, there is still no specific antidote even after 125 years of research and use. Ingestion of >20 ml of a 20% preparation is likely to cause death from multi-organ failure and cardiogenic shock within 1 to 4 days, while smaller quantities (10–20 ml) may initiate an irreversible lung fibrosis and kidney failure resulting in death within several weeks.^[4]^ As the half-life of paraquat is very long, the mortality rate is extremely high in patients with acute kidney dysfunction.^[5]^ Sato^[6]^ pointed out that patients whose urine paraquat levels are higher than 10 mg/ml or urine dithionate test showed blue color within 24 hours of ingestion would most likely die. Although the oral dose of this patient was not exact, urine dithionate test showed light blue color and blood paraquat concentration was 0.547 μg/ml at the 10th hour following ingestion which is suggestive of severe poisoning and prognosis. Besides, the hallmark of paraquat poisoning is the generation of free oxygen radicals which cause tissue and organ damage.^[7]^ Therefore, metabolic acidosis, liver, and kidney dysfunction are considered to be fatal.^[4–8]^ For this patient, acidosis rapidly increased and creatinine with ALT and TBIL rose after admission which were consistent with her final outcome.

Theoretically, the molecular weight of paraquat aqueous solution is only 186.256, which is relatively small and does not combine with plasma proteins. These biochemical characteristics suggest that paraquat could pass through the placenta. Animal experiments^[9]^ on C^[14]^ isotope labeled paraquat in rats and rabbits have confirmed this. Moreover, the ability of paraquat entering the fetus via the placenta can be observed in the late second trimester of rhesus macaque pregnancy using positron emission computed tomography (PET/CT) imaging.^[10]^ Previously, the paraquat level in infants was reported to be higher than in the mother,^[11]^ although, in our case the levels were approximately the same. This may be due to the different detection time or gestational weeks in our case report compared to the earlier reported case. Thus, all of the above present evidence to suggest that paraquat can enter the fetus via the placenta. Apart from the lung, fetal urine is the main source of amniotic fluid in late pregnancy. Fluid exchange between amniotic fluid and fetus is accomplished mainly through the digestive, urinary, and respiratory tracts. The respiratory distress and tongue and oral mucosal ulcer after birth in this infant can also be explained by the above mechanisms. Moreover, the concentration of paraquat in amniotic fluid in this case was similar to concentrations in the mother's and infant's blood. This makes us speculate that the fetal organ function, especially the kidney, liver, and lung function, develops profoundly in the late pregnancy, and the concentration is balanced among these3.

Paraquat poisoning can lead to severe multiple organ dysfunction and pathological changes in pregnant women. Autopsy has showed that^[12]^ the lung spindle-shaped cells and multinucleated giant cells proliferated, and the cell membrane became thicker causing alveolar congestion and pulmonary consolidation in a 28 weeks pregnant woman who had experienced paraquat poisoning. In that case also, there was some fatty infiltration in the centrilobular areas of the liver and patchy necrosis in the proximal tubules of kidney. However, nothing had been found in the fetus. This is probably a reflection of absent pulmonary function and minimal kidney tubular function of the fetus at that stage. In our case, the serum chemistry suggested liver and kidney dysfunction, but we could not perform an autopsy to determine the organ damage in later gestational age. Moreover, there were several low echoes in the parenchyma of placenta in this case as found in preoperative B ultrasound and focal fibrinous necrosis in the placenta. It is possible that they were evidence of placental infarction as previously reported.^[13]^

Fetal mortality after maternal paraquat poisoning is extremely high and cases of survival are rare.^[11–13,14]^ Satariya^[15]^ had reported 4 deaths in 5 infants in the third trimester and 3 deaths in 13 infants whose mothers developed paraquat systemic toxicity during pregnancy, while 14 of 23 infants survived in mothers without a systemic effect. The judgment of fetal outcome is helpful for obstetricians to determine the termination of pregnancy risk in pregnant women with paraquat poisoning. However, this is also an ethical issue. Since, there is no literature report on the exact timing of caesarean section yet, we believe that it is important to judge the fetal maturity accurately to determine the treatment of the fetus. In this case, the patient was pregnant for 38^+^ weeks with pernicious placenta previa and the fetus was mature; thus, caesarean section and hysterectomy were considered appropriate. In addition, initiative hemoperfusion together with pulse dexamethasone therapy are also important. However, the course and manifestation of organ toxicity in new-born infants will closely mimic that of the mother in most cases. Maternal and fetal outcomes are still mainly associated with oral dose and early treatments.

## 6 Conclusion

Paraquat can enter the fetus through the placenta in acute poisoning or can transfer to the amniotic fluid by fluid exchange between mother and fetus. The pathological changes of fetal organs after paraquat poisoning may relate to the gestational age, which means the higher the gestational age, the more significant the changes. Although the prognosis is very poor, choosing comprehensive treatment and appropriate timing of treatment may benefit both the mother and fetus.

## Author contributions

**Conceptualization:** Guangcai Yu.

**Investigation:** Jianshi Chen, Min Si.

**Project administration:** Xiangdong Jian.

**Resources:** Jianshi Chen, Xiangdong Jian.

**Writing – original draft:** Jianshi Chen.

**Writing – review & editing:** Guangcai Yu, Baotian Kan.
